# Hijacking Host Immunity by the Human T-Cell Leukemia Virus Type-1: Implications for Therapeutic and Preventive Vaccines

**DOI:** 10.3390/v14102084

**Published:** 2022-09-20

**Authors:** Cynthia A. Pise-Masison, Genoveffa Franchini

**Affiliations:** Animal Models and Retroviral Vaccines Section, Center for Cancer Research, National Cancer Institute, Bethesda, MD 20892, USA

**Keywords:** human T-cell leukemia virus, HTLV-1, adult T-cell leukemia/lymphoma, ATLL, HTLV-1-associated myelopathy/tropical spastic paraparesis, HAM/TSP, vaccine, immunology

## Abstract

Human T-cell Leukemia virus type-1 (HTLV-1) causes adult T-cell leukemia/lymphoma (ATLL), HTLV-1-associated myelopathy/tropical spastic paraparesis (HAM/TSP) and other inflammatory diseases. High viral DNA burden (VL) in peripheral blood mononuclear cells is a documented risk factor for ATLL and HAM/TSP, and patients with HAM/TSP have a higher VL in cerebrospinal fluid than in peripheral blood. VL alone is not sufficient to differentiate symptomatic patients from healthy carriers, suggesting the importance of other factors, including host immune response. HTLV-1 infection is life-long; CD4^+^-infected cells are not eradicated by the immune response because HTLV-1 inhibits the function of dendritic cells, monocytes, Natural Killer cells, and adaptive cytotoxic CD8^+^ responses. Although the majority of infected CD4^+^ T-cells adopt a resting phenotype, antigen stimulation may result in bursts of viral expression. The antigen-dependent “on-off” viral expression creates “conditional latency” that when combined with ineffective host responses precludes virus eradication. Epidemiological and clinical data suggest that the continuous attempt of the host immunity to eliminate infected cells results in chronic immune activation that can be further exacerbated by co-morbidities, resulting in the development of severe disease. We review cell and animal model studies that uncovered mechanisms used by HTLV-1 to usurp and/or counteract host immunity.

## 1. Introduction

Human T-cell leukemia/lymphoma virus type-1 (HTLV-1) is the first pathogenic retrovirus discovered in humans [1,2]. Its current prevalence is unknown, with estimates ranging from 10 to 20 million people worldwide [3]. While the majority of HTLV-1-infected individuals remain asymptomatic, after a long period of clinical latency a low percentage of patients develop either adult T-cell leukemia/lymphoma (ATLL), a disease characterized by malignant proliferation of CD4^+^ T-lymphocytes, or HTLV-1-associated myelopathy/ tropical spastic paraparesis (HAM/TSP), a neurodegenerative condition of possible auto-immune nature [4,5,6,7,8,9,10,11,12,13]. HTLV-1 is also associated with other clinical disorders including HTLV-1-associated arthropathy, HTLV-1-associated uveitis, infective dermatitis, polymyositis, and bronchiolitis [14,15,16]. To date, no disease-specific differences in viral strains have been identified, and it appears that the chronic inflammation associated with HTLV-1 infection may be at the basis of diseases manifesting as lymphoproliferation and degenerative inflammatory diseases. Although some progress has been made in therapies for these diseases, the prognosis for ATLL is still dismal, and HAM/TSP remains an intractable disease. The aim of this review is to provide an overview of the current state of knowledge of the interplay between HTLV-1 and host immunity.

## 2. HTLV-1 Transmission

The genomic organization and nucleotide sequence of HTLV-1 isolates are highly conserved. To classify HTLV-1 into different subtypes (subtypes A-G) with characteristic geographic distribution, variations in the sequence of the HTLV-1 long terminal repeat (LTR) sections have been used [17]. The predominant subtype in central Australia is HTLV-1C. In some regions of Australia, HTLV-1C has an extremely high prevalence of approximately 30% infection among indigenous populations, representing a public health emergency. As well as a risk of developing ATLL and HAM/TSP, HTLV-1C infected individuals have elevated mortality and develop lung inflammation, bronchiectasis and infectious diseases at an increased frequency [16]. Sequence analysis found that HTLV-1C is most divergent from the other HTLV-1 subtypes at the 3′ section of its genome. Whether these differences truly contribute to differences in viral pathogenicity or are due to virus-host co-evolution is not yet known [18]. 

HTLV-1 infection occurs primarily through cell-to-cell contact between the virus-infected CD4^+^ T-cell and uninfected cells. The most common routes of transmission are mother-to-infant, sexual intercourse (mainly male-to-female), and, rarely, blood transfusion (whole blood products and sharing syringes) and organ transplants [19,20]. Cell-free HTLV-1 infection has not been documented. 

Risk factors of HTLV-1 vertical transmission are mainly associated with breastfeeding and additional factors, such as vulnerable socioeconomic position, with the rate of vertical transmission ranging from 3.9% to 22% in endemic areas [21]. Of note, vertical transmission has been associated with diseases such as uveitis and ATLL.

Another major route of transmission is sexual intercourse in both genders, as HTLV-1-infected cells are present in genital secretions, such as vaginal mucus or secretions and semen [22]. Many infected cells are found in semen, perhaps accounting for more effective male-to-female and male-to-male transmission [23]. Studies from Japan showed that the male-to-female transmission rate of HTLV-1 was 60.8%, but female-to-male transmission was only around 0.4% [24].

Increased HTLV-1 transmission may occur in individuals infected with other sexually transmitted diseases because these infections induce inflammatory reactions that recruit lymphocytes, which have a high proportion of CD4^+^ T-cells facilitating HTLV-1 transmission [25]. In addition, several factors such as age over 45 years old, menopause, and a high number of HTLV-1-infected cells can increase the number of HTLV-1 positive cells in the seminal and vaginal fluids [23], increasing HTLV-1 transmission risk. Increased risk of viral transmission has been associated with the presence of neutralizing antibodies against Tax. A 1991 study demonstrated that 75% of HTLV-1-infected males had antibodies against Tax [26]. It is possible that the level of Tax neutralizing Abs reflects a more active viral replication in vivo in males favoring virus transmission to females.

HTLV-1 transmission can occur through whole blood transfusions or white blood cell contaminated plasma [27,28,29]. Intravenous exposure to HTLV-1-infected blood leads to seroconversion in 40–60% of cases [30]. It is estimated that the transfer of ≥9 × 10^4^ HTLV-1-infected cells is necessary to establish a transfusion-transmitted HTLV-1 infection [31]. Sharing needles among intravenous drug abusers can also transfer infected cells [32,33,34,35]. 

HTLV-1 transmission also may occur during allograft transplantation [36]. Despite low incidence, myelopathy cases have been reported following organ transplantation in HTLV-1 positive subjects in non-endemic countries [37]. In 2000, three patients who received organ transplants from the same donor, who was determined to be an HTLV-1 healthy carrier, then presented with clinical manifestations of myelopathy [38]. The development of HAM/TSP in recipients from HTLV-1 healthy carriers has been reported and can be rapid and progressive [30,39,40]. In the HTLV-1 endemic regions, more cases of HAM/TSP and ATLL subjects have been reported after allograft transplantation from HTLV-1 carriers [41,42]. It appears that the immunosuppression used to avoid organ rejection is a primary factor in frequent and rapid disease onset [20]. Alternative explanations include high doses of virus exposure due to the large numbers of infected cells in the contaminated organs. 

Although HTVL-1 can infect various cell types, such as dendritic cells, B cells, macrophages and T-cells, the virus preferentially induces clonal expansion of CD4^+^ T-cells [43,44] and has an impact on T-cell function contributing to disease progression [45,46,47]. Although in vitro cell-free virus transmission has been demonstrated for DCs and monocytic cell lines [48,49], HTLV-1 is believed to be transmitted to T-cells and myeloid cells primarily by cell-to-cell contact through virological synapse, biofilm-like extracellular viral assemblies or cellular conduits [50,51,52]. Viral genes are responsible for clonal proliferation of infected cells, de novo infection, and infected cell survival. Importantly, viral gene expression is also critical for the virus’s ability to evade the host immune response.

## 3. Immune Deregulation in HTLV-1 Infection

HTLV-1 infection is associated with diseases that are often accompanied by changes in immune responses (4–16). ATLL is often associated with severe immune suppression, while HAM/TSP is accompanied by chronic inflammation. CD4 cells regulate immune responses, but due to viral infection, their function is altered, causing changes in inflammatory responses and immune tolerance. Increased Treg cell function and production of IL-10 and TGF-b trigger the immunosuppressive phenotype observed in patients [53,54]. In HAM/TSP patients, unlike in ATLL, investigators found decreased FoxP3 expression and reduced IL-10 and TGF-b [55]. The loss of suppressive function may cause chronic inflammation, T-cell and Natural Killer (NK) cell exhaustion and exacerbate the disease process. HTLV-1-infected CD4^+^ cells of HAM/TSP patients exhibit spontaneous proliferation with an increased production of proinflammatory cytokines such as interferon (IFN)-γ, TNF-α, IL-1 and IL-16 and neurotoxic cytokines IFN-γ and TNF-α, which are found in high concentrations in the spinal fluid of HAM/TSP patients [20,56,57]. Disruption of the cytokine homeostasis and the balance between inflammatory and anti-inflammatory responses is thought to lead to loss of tolerance and the development of autoimmunity.

The type-I interferon response is induced by viral infection [58,59,60,61,62]. The culture of HTLV-1-infected cells with IFNs suppress HTLV-1 expression [58]. HTLV-1 mRNA and protein expression are markedly decreased when infected cells are co-cultured with stromal cells through type-I IFN responses [62]. Furthermore, it was shown that HTLV-1 infection reduces the phosphorylation of factors in the IFN signaling cascade [59], and that the viral proteins Tax and p30 can regulate cellular transcription factors such as SOCS1 and PU.1, which inhibit the interferon response [48,60,63,64,65]. Interestingly, the combination of the antiviral drugs zidovudine (AZT) and IFN-α have become the standard treatment of some forms of ATLL and significantly improves survival for patients diagnosed with chronic or smoldering subtypes, or a portion of acute individuals carrying wild-type p53 [66,67,68,69]. These data suggest that the antiviral effect of both drugs may target an ongoing, low level of viral infection/replication.

## 4. Genomic Organization

After entry of the virus into a host cell, the viral RNA is reverse transcribed into double-stranded DNA, which integrates into the host chromosomal DNA and results in lifelong infection. The HTLV-1 integrated genome (provirus) contains the characteristic retroviral structural and enzymatic genes *gag*, *pro*, *pol*, and *env* [7]. In addition, a region located between *env* and the 3′ long terminal repeat (LTR), contains four partially overlapping open reading frames (*orf*s) expressing regulatory proteins [7] that are produced via alternatively spliced *mRNAs* and by internal initiation codons [70,71,72,73]. *Orf-I* produces the p12 protein, which is proteolytically cleaved at the amino terminus to generate the p8 protein, while differential splicing of mRNA from *orf-II* results in production of the p13 and p30 proteins [71,73,74,75]. The HTLV-1 regulatory genes p12, p8, p30, and p13 are not absolutely required for virus replication or immortalization of human primary T-cells in vitro [76,77,78]. Nevertheless, several studies have shown that primary human T-cells immortalized with molecular clones lacking p12 or p30 grew less efficiently than the wild type molecular clone and are more dependent on IL-2 [78,79,80].

Interestingly, it was shown that the HTLV-1C subtype does not encode the *orf-I* gene [81]. In HTLV-1C proviral sequences from 22 Australian isolates, a mutation at position 6840 leads to a change of the start codon for *orf-1* from methionine to a threonine [82]. Although the p12/p8 protein would not be expressed, a bicistronic mRNA, *rex*-*orf*-*I*, uses an initiation codon in exon 2 and the acceptor splice site at position 6383 to encode a protein of 152 amino acids referred to as the Rex-orf-I protein of 17 kDa. In this mRNA, the first coding exon of the Rex protein is joined in frame to p12/p8. The distinct functional motifs implicated in p12 function are conserved in the amino acid sequence of the putative protein *rex*-*orf*-*I*, and thus could possibly compensate for the role of p12 in viral persistence and immune dysregulation. 

*Orf-III* and *orf-IV* encode for the Rex and Tax proteins that are essential for viral expression and production, respectively, and an antisense mRNA transcribed from the 3′ LTR that generates the HTLV-1 basic leucine zipper (HBZ) protein [83,84,85,86]. All regulatory proteins interfere with cellular pathways and only Tax and Rex are essential for virus expression and production in vitro and likely in vivo. The regulatory proteins p12/p8, p30, and HBZ are dispensable for viral replication in vitro but essential for viral persistence in vivo (see next sections). To date, there is no disease-specific difference in viral strains, and it is unclear how infection results in asymptomatic, cancer, neurodegenerative or inflammatory diseases. It is thought that the viral regulatory proteins play an important role in pathogenesis.

## 5. Tax and HBZ-Specific Cytotoxic Response and Viral Burden

The prognosis for ATLL is still bleak and HAM/TSP remains an intractable disease. The two regulatory proteins of HTLV-1, Tax, and the *HTLV-1 bZIP factor*, HBZ, have been shown to have pleiotropic functions connected to viral pathogenesis. Many early studies focused on the viral transcriptional activator, Tax. In addition to being required for induction of the 5′ viral long terminal repeat, and thus the expression of viral sense strand genes, Tax has been shown to regulate the expression of NF-kB- and CREB-responsive genes, cellular pathways central to immunity [87,88]. Tax has also been shown to have cell-dependent pro- or anti- apoptotic activity and to affect DNA repair [89,90,91,92,93,94]. Therefore, Tax is thought to play a major role in the proliferation of infected cells, as well as in inducing genomic instability, thereby contributing to viral oncogenesis. NF-kB regulates physiological processes such as proliferation, cell death, inflammation, and immunity [87], and has been shown to be constitutively activated in HTLV-1-infected cells. Therefore, it is believed that NF-kB activation is central for HTLV-1 associated inflammation and cancer. 

However, while Tax expression is high in early infection, it is often suppressed at later timepoints, likely because Tax is highly immunogenic and renders infected cells vulnerable to cytotoxic T-cells [93,95,96,97]. Tax expression is suppressed transcriptionally by HBZ and post-transcriptionally by both p13 and p30 [98,99]. p30 has been found to regulate Tax and Rex expression and viral production by sequestering the common *Tax/Rex* doubly-spliced RNA in the nucleus [100], whereas p13 binds Tax and interferes with its activity [101]. Other mechanisms identified to inhibit Tax expression include mutations in the tax gene [102], methylation or deletion of the 5′ LTR, and host restriction factor CTIIA [103,104,105]. The transcription factor CTIIA, which regulates major histocompatibility complex (MHC) class II expression, was shown to bind Tax and reduce its activation of viral transcription [105]. Interestingly, Tax has been shown to increase MHC-II basal expression by interacting with NF-YB [106]. However, more studies are needed to determine the possible interplay between Tax and CTIIA on MHC-II expression and its impact on peptide presentation. 

Recent ex vivo studies in T-cell clones showed that Tax can be expressed in bursts [107] that can be triggered by cellular stress [108] and can toggle between an on and off state [100,101,109,110,111]. While Tax is often silenced in the later stages of infection, HBZ, encoded by the minus strand HTLV-1 RNA, is constitutively expressed at very low levels in vivo throughout infection [85]. HBZ has been shown to have a variety of functions that are thought to play a role in viral persistence and pathogenesis [47,90,110]. Interestingly, HBZ has been shown to counter many of the activities of Tax. Recently, cytoplasmic versus nuclear localization of the HBZ protein has been shown to differ in asymptomatic carriers and HAM/TSP patients compared to ATLL patients, in the distribution of the HBZ protein in peripheral blood mononuclear cells [112]. It has been shown that not only HBZ protein but also HBZ mRNA, which is retained in the nucleus, may be involved in HLTV-1 mediated cell proliferation and anti-apoptosis [85,113]. Unlike Tax, the cytotoxic T lymphocyte (CTL) response to HBZ is very low [114,115]. It remains unclear, however, whether the low immunogenicity is intrinsic to HBZ or is linked to its low expression in vivo. 

The CTL response is a critical component of the host immune response against viral infection. CTLs directed toward HTLV-1 predominantly recognize the Tax antigen, and anti-Tax CTLs have been suggested to contribute to the control of expansion of infected cells [95,116,117,118,119,120,121]. Similarly, even if the immunogenicity of HBZ is low [115], correlative analyses suggest that CTL responses to HBZ may contribute to the control of virus burden [114,122,123]. However, all these studies are correlative and performed either on ex vivo tetramer stained cells or stimulated cells apart from their natural micro-environment. Several studies have demonstrated a functional impairment of ex vivo CTL in HAM/TSP linked to the exhaustion associated with chronic immune activation. While direct evidence that CTL controls the HTLV-1 viral burden is lacking in humans, CD8^+^ T-cell depletion, as a means to demonstrate their importance in non-human primates, has demonstrated that their decrease accelerates primary HTLV-1 infection [124].

## 6. HTLV-1 Regulatory Genes

### 6.1. The Pleiotropic orf-I Encoded p12/p8 Proteins

HTLV-1 *orf-I* encodes a 99 amino acid p12 protein which can be proteolytically cleaved at the amino terminus to generate the p8 protein [74]. The two protein isoforms localize to different cellular compartments and are associated with infected cell proliferation, as well as the ability of the virus to evade several arms of immunity such as cytotoxic T-cells, NK cells, and monocyte efferocytosis. *Orf-I* mRNA is expressed early after virus entry and is critical for establishing and maintaining viral infection in vivo [78,125,126,127].

### 6.2. The p12 Protein in the ER

#### 6.2.1. T-Cell Proliferation

HTLV-1 persists primarily through the proliferation of infected cells. The viral p12 protein localizes to the endoplasmic reticulum (ER) through a noncanonical ER retention signal [75]. In the ER, p12, through its interaction with calcium binding proteins calnexin and calreticulin, increases cytosolic calcium [128]. In T-lymphocytes, the increased ER calcium release is mediated by inositol triphosphate receptors. In response to the lower level of calcium in the ER, calcium enters through calcium channels in the plasma membrane [129,130]. By depleting ER calcium stores and increasing cytosolic calcium, p12 modulates a variety of processes including T-cell proliferation, viral replication, and viral spread. Early studies demonstrated that overexpression of *orf-I* influenced T-cell proliferation by activating the nuclear factor of activated T-cells (NFAT), which is dependent on calcium-binding proteins for its dephosphorylation and nuclear import, to increase T-cell proliferation [129,130,131]. During the immune response, NFAT activation is controlled by calcium influx upon T-cell activation. Recognition of specific peptide-bound MHC molecules by the T-cell receptor (TCR) activates a cascade of events that lead to NFAT activation. Upon ligand binding, the protein tyrosine kinases Lck and Fyn phosphorylate the TCRζ and CD3 subunits, allowing ZAP70 docking and activation. ZAP70 then phosphorylates the linker of activation of T-cells (LAT) that, in turn, binds and activates phospholipase C-γ-1 (PLCγ1). This leads to the production of inositol-1,4,5-trisphosphate and the release of ER calcium stores. The increase in intracellular calcium stimulates NFAT dephosphorylate by the Ca^2+^/calmodulin-dependent phosphatase calcineurin, triggering NFAT’s nuclear import. Because p12 can modulate the cytosolic calcium levels, it can also activate NFAT independent of TCR signaling [129]. NFAT is known to bind to and activate transcription of the IL-2 promoter, and thus p12 can increase the production of IL-2 in T-cells in a calcium-dependent process [130]. The expression of p12 can also modulate other calcium-regulated proteins such as p300, a transcriptional coactivator [132]. Since p300 is known to play a role in Tax-mediated LTR activation, this suggests that p12 may aid in viral gene expression [133]. In a calcium dependent manner, p12 may enhance intercellular viral transmission by inducing the cellular adhesion through the clustering of Lymphocyte Function Associated Antigen 1 (LFA-1) on the surface of T-cells, which is known to promote cell-to-cell contacts [134]. 

In addition, early studies demonstrated another function in the ER of p12. P12 binds to the IL-2R β chain in a region critical for JAK1 and JAK3 recruitment, and the interaction of p12/p8 with the immature IL-2R leads to an increase in Signal Transducer and Activator of Transcription 5 (STAT5) phosphorylation and DNA binding activity and decreases the cellular requirement for IL-2 [79]. Furthermore, the binding of p12 to IL-2R allows T-cells to proliferate not only with a lesser amount of IL-2, but also with suboptimal antigen stimulation, providing a proliferative advantage to HTLV-1-infected cells [79]. 

#### 6.2.2. MHC-Class I and Cytotoxic T-Cells

The presentation of antigens via the MHC class I (MHC-I) processing pathway plays a critical role in the development of host immunity against pathogens. All nucleated cells express MHC-I on their cell surface. MHC-I molecules present antigen peptides to the TCRs on effector CD8^+^ T-cells, also called cytotoxic T lymphocytes (CTLs). Because CTLs recognize viral peptide:MHC-I complexes on target cells, many viruses have evolved proteins to interfere with this pathway [135]. The MHC-I molecule is composed of a heavy chain (Hc) that is non-covalently bound to a nonglycosylated β2 microglobulin protein (β2M). The affinity of the MHC-I heavy chain is increased in the presence of peptide and folds to assemble the peptide:MHC-I-Hc: β2M complex in the ER lumen [136]. Early work showed that, prior to association with β2M, the p12 protein binds to newly synthesized MHC-I-Hc, preventing its maturation [137]. These improperly assembled protein complexes are cleared from the ER by degradation [138]. Immature MHC-I-Hc:p12 complexes are ubiquitinated, retro-translocated to the cytoplasm, and degraded by the proteasome, resulting in decreased MHC-I surface expression [137]. Although the viral p8 protein was also able to bind MHC-I, its biological importance has not been investigated. Interestingly, a study comparing MHC-I expression on the surface of primary CD4^+^ T-cells infected with HTLV-1 mutant viruses (HTLV-1_WT_, HTLV-1_G29S_, HTLV-1_N26_, HTLV-1_p12KO_) demonstrated that a decrease in surface MHC-I was seen only in cells infected with virus that predominantly expresses the p12 protein HTLV-1_G29S_ [139]. This same study showed that expression of p12 and p8 (HTLV-1_WT_) was necessary for the protection of infected CD4^+^ cells from CTL lysis [139]. By preventing the presentation of viral antigens through the MHC-I presentation pathway, p12/p8 may contribute to the expansion of infected T-cell clones by allowing the evasion of the adaptive immune surveillance in vivo.

#### 6.2.3. ICAM-1 and ICAM-2 and NK

NK cells detect and destroy cells expressing low surface MHC-I levels. Thus, reduced MHC-I cell-surface expression enables infected cells to evade CTL killing but makes them targets for NK cells. NK cells directly kill target cells by delivering cytotoxic proteins (perforin and granzyme B) to their targets. When NK cells recognize a target, a lytic immune synapse is established through integrins like LFA-1 on the NK cell, and its ligand intercellular adhesion molecule 1 (ICAM-1) on the target cell [140]. Early studies demonstrated that overexpression of Tax induced surface expression of the adhesion molecules LFA-3 and ICAM-1 [141,142]. Although ICAM-1 levels were high on Tax-expressing HTLV-1 transformed cell lines, it was found that the expression of its ligand LFA-1 was independent of HTLV-1 infection, and was low in three of four ATL cell lines [142]. Later studies found that the surface expression of MHC-I, ICAM-1, and ICAM-2, but not ICAM-3, was significantly reduced in HTLV-1-infected primary CD4^+^ T-cells, making them resistant to autologous NK cell killing [143]. Pretreatment of the NK cells with IL-2 only marginally increased their ability to kill infected cells. In addition to reduced MHC-I and ICAM-1/2, HTLV-1-infected CD4^+^ T-cells did not express ligands for NK cell activating receptors NCR and NKG2D, further contributing to the reduced adherence of NK cells to HTLV-1-infected cells [143]. This study went on to show that expression of p12^I^ in primary CD4^+^ T-cells was sufficient to cause downregulation of surface ICAM-1 and ICAM-2. 

The immunomodulatory drug Pomalidomide (Pom), used as part of the standard treatment for multiple myeloma and recently approved for the treatment of Kaposi Sarcoma [144,145], increased both MHC-I and ICAM-1 on Tax-expressing cells. The treatment of HTLV-1-infected cells with Pom increased surface expression of MHC-I, ICAM-1, and B7-2 and significantly increased the susceptibility of infected cells to NK cell killing. Furthermore, the effect of Pom was dependent on *orf-I* expression, as the surface expression of both MHC-I and ICAM-1 increased following Pom treatment in primary CD4^+^ cells infected with wild type HTLV-1 but not primary CD4^+^ cells infected with a mutant *orf-I* knockout HTLV-1 virus [146]. Additional studies demonstrated that the thalidomide drugs Pom and the related analogue lenalidomide (Len) directly affected HTLV-1-infected cell proliferation by reducing the transcription factors involved in cell signaling and survival: IRF4, STAT3, EZH2, Aiolos and Ikaros [146,147,148]. Thus, Pom treatment could potentially reduce the viral burden in HTLV-1-infected individuals by rendering them susceptible to CTL and NK cell killing. Indeed, the importance of NK and CTL cells in controlling infection is underscored by macaque studies in which the depletion of CD8^+^ cells greatly enhanced the infection of both wild type and *orf-I* knockout virus [124]. Although Pom treatment of HTLV-1-infected macaques did result in the activation of T-cells, this immune activation was transient and viral activation was also found [149]. While a phase II trial of lenalidomide in the United States of four patients with refractory/relapse ATLL had no clinical activity, Len did have tolerable toxicity and provided significant anti-cancer activity in a phase II clinical trial in Japan of 26 relapsed/recurrent patients (15 acute and four chronic cases of ATL and seven cases of lymphoma) [150,151]. These results have led to the approval of Len for the treatment of refractory/relapse ATLL in Japan.

### 6.3. The p8 Protein in T-Cells and Monocytes

#### 6.3.1. The p8 Protein and the TCR

T-cells are critical in mediating the protective immune response to pathogens. The localization of p8 on the surface of T-cells was shown to decrease T-cell activation by inhibiting proximal T-Cell Receptor (TCR) signaling [152]. The recognition of peptide-bound major histocompatibility complex II (MHC-II) on antigen-presenting cells via the TCR induces TCR ligation and recruitment of the complex to lipid rafts and the immunological synapse (IS). The p8 protein also localizes to the IS upon TCR ligation, causing a LAT-dependent decrease in phosphorylation of LAT, VAV and PLCγ1, downregulating NFAT activation [74,152]. Thus, p8 is able to impair antigen-specific T-cell responses to immunologic stimuli, a state called T-cell anergy. Induction of T-cell anergy by p8 was shown to result in decreased Tax activity and thus decreased viral replication [152]. However, because p8 is known to be transferred to target cells through cellular conduits, p8-induced T-cell anergy in neighboring cells may increase viral transmission [51,153]. 

#### 6.3.2. The p8 Protein and Viral Transmission

It is well-documented that HTLV-1 is transmitted via cell-to-cell contact and that cell-free virus is poorly infectious and rarely detected in the blood plasma of HTLV-1-infected individuals [49,154,155,156]. Three modes of cell-to-cell viral transmission have been identified: the virological synapse, biofilm-like extracellular viral assemblies, and cellular conduits [50,51,52,157]. Virus transmission through the virological synapse depends on the polarization of cytoskeletal and adhesion molecules to the cellular contact [50]. Cellular surface adhesion molecules are also important for viral transmission. The HTLV-1 p8 protein enhances LFA-1 clustering on the cell surface, increasing cell-to-cell contacts and poly synapse formation, which promotes viral transfer [51,134]. The p8 protein also promotes the formation of cellular conduits, thin membranous protrusions used by several different cell types for intercellular communication [51,158,159]. Immune cells such as macrophages, B cells, NK cells and T-cells are known to use tunneling nanotubes (TNTs) for intercellular communication [160,161]. TNTs are filamentous actin containing structures that function as long cytoplasmic bridges connecting adjacent or distant cells for efficient cell-to-cell communication. The p8 protein was shown to induce TNT formation, increasing the quantity and length, and allowing the transfer of HTLV-1 proteins such as Tax, Gag, Envelope, and p8 itself [51]. Other viruses have been shown to induce TNTs to enhance viral spread and avoid immune recognition [162,163,164,165,166]. When HTLV-1-infected T-cells are treated with Cytarabine, a molecule shown to reduce TNT formation [167], virus transmission is decreased by 30% [168]. Furthermore, using a quantitative flow cytometry method, the p8 protein was shown to be transferred to approximately 5% of recipient T-cells after 5 min of co-culture in a process dependent on actin polymerization [51,168,169].

#### 6.3.3. The p8 Protein and VASP

Interestingly, the vasodilator-stimulated phosphoprotein (VASP), which promotes actin filament elongation, co-immunoprecipitated with p8, and imaging showed partial areas of co-localization of VASP and p8 on the plasma membrane and in membrane protrusions [153]. The knockdown of VASP expression by RNA interference or CRISPR/Cas9 reduced p8 and Gag transfer to target cells, but virus release was unaffected [169]. Since VASP is associated with filamentous actin formation, it likely plays a widespread role in cell adhesion and motility, and contributes to intracellular signaling pathways that regulate integrin-extracellular matrix interactions, as well as processes dependent on cytoskeleton remodeling and cell polarity such as T-cell activation and phagocytosis [170].

#### 6.3.4. The p8 Protein and Monocytes

The role of p12/p8 in monocyte function is unclear. It was shown that HTLV-1 virus knocked-out for Orf-I protein expression was severely impaired in its ability to replicate in dendritic cells [126]. Furthermore, when mutant viruses were used to infect the monocytic cell line THP-1, we found that p8 expressing virus (HTLV-1_N26_) infected monocytes similar to wild type virus, with a proviral load of three to four copies per cell and high supernatant p19 levels. In contrast, mutant viruses expressing only p12 (HTLV-1_G29S_) or no p12/p8 (HTLV-1_p12KO_) had lower proviral loads of > one copy per cell and no detectable supernatant p19 produced [139]. This is similar to what we found in the rhesus macaque model, where HTLV-1_G29S_ and HTLV-1_p12KO_ did not establish persistent infection, while HTLV-1_WT_ and HTLV-1_N26_ did [139]. Orf-I also alters the engulfment of infected cells by monocytes. In vitro experiments in human primary monocytes or THP-1 cells demonstrated that *orf-I* expression is associated with the inhibition of inflammasome activation, with increased CD47 “don’t-eat-me” signal surface expression in virus-infected cells and the decreased monocyte engulfment of infected cells [124]. 

#### 6.3.5. p12/p8 and Vacuolar ATPase

Similar to the E5 protein of the bovine papilloma virus, both p12 and p8 can bind to the proton pump V-ATPase through the 16 kilodalton subunit [171,172,173,174]. V-ATPase localizes to and regulates the acidification of intracellular vesicles such as clathrin coated vesicles, endosomes, lysosomes, Golgi vesicles, endoplasmic reticula, and synaptic vesicles [175]. The binding of the V-ATPase with the HTLV-1 p12 and p8 proteins may potentially interfere with functions such as protein trafficking within the lysosomal/endosomal vesicles or the dissociation of receptor-ligand complexes, but acidification of intermediates between early and late endosomes or endosome carrier vesicles remains essential [176,177]. HTLV-1 is known to infect dendritic cells and monocytes/macrophages where acidification of lysosomes may regulate virus entry or egress [49,178,179], and monocyte functions such as phagocytosis and efferocytosis. Of note, the knocking out of orf-I expression impairs HTLV-1 persistence in dendritic cells [126] and affects efferocytosis.

### 6.4. The Pleiotropic orf-II Encoded p30 and p13 Proteins

The *orf-II* gene encodes for two proteins: p30, a 241-residue nuclear/nucleolar protein expressed from a doubly-spliced mRNA, and p13, an 87-residue protein coded by a singly-spliced mRNA corresponding to the carboxy-terminal portion of p30 [71,73,75]. HTLV-1 can infect monocytes/macrophages and dendritic cells [49,180,181,182,183,184,185,186,187], but their role in viral pathogenesis is not fully understood. While the majority of viral DNA in infected individuals is found in CD4^+^ and CD8^+^ T-cells, a small percentage is observed in all three monocyte subsets defined by CD14 and CD16 expression [44], suggesting that they might be involved in the pathogenesis of the virus. 

#### 6.4.1. p30 Protein Modulates the Interferon Response

Interferons (IFN-Iα and IFN-Iβ) play a critical role in mediating innate and adaptive antiviral immunity. This is accomplished predominantly through their impact on cell activation, cell proliferation, and apoptosis. Activation of the IFN response increases the expression of over 300 genes encoding antiviral and immunoregulatory proteins [186,188,189,190,191]. IFNs are primarily produced by dendritic cells, fibroblasts, and macrophages. Dendritic cells isolated from HTLV-infected individuals were found to have reduced IFN secretion, suggesting that the virus has strategies to escape the interferon response [186]. Consistent with impaired IFN responses, reduced phosphorylation of members of the IFN cascade (TYK2 and STAT2) were observed in HTLV-1 positive cells [92,192,193,194,195]. In addition, STAT1 phosphorylation, most likely mediated through the STAT1 negative regulator, was suppressed in ex vivo CD4^+^ T-cells isolated from HTLV-1-infected patients [64,196].

Early studies demonstrated that the HTLV-1 p30 protein could work as a latency factor by retaining newly transcribed *tax/rex* mRNA in the nucleus, as well as by repressing LTR-mediated transcription [100,111]. It was later demonstrated that in monocytic cells, p30 affects Toll-like receptor signaling and cytokine release [48,63]. TLRs are an important defense against microbial pathogens. Because TLR activation is crucial for dendritic cell maturation, TLRs link innate and pathogen-specific adaptive responses. TLR3, TLR4, TLR7, TLR8, and TLR9 activation can induce an antiviral response by inducing type I IFNs [197,198,199]. The p30 protein, through direct interaction with the transcription factor PU.1, was shown to reduce cell surface expression of TLR4 [63]. In addition, it was further shown that p30 decreases PU.1 recruitment to IFN-responsive gene promoters following stimulation by either lipopolysaccharide (LPS) or poly(IC), which respectively activate the toll-like receptors TLR4 and TLR3 [48]. Following LPS stimulation of monocytes/macrophages, reduced TLR4 expression resulted in the reduced release of MCP1, TNF-α, and IL-8 (proinflammatory cytokines), and an increased release of the anti-inflammatory cytokine, IL-10 [63]. Consistent with p30 affecting cytokine release, high levels of IL-10 secretion from HTLV-1-infected cell lines and in the plasma of patients with ATLL have been documented [200,201]. The inhibitory effect of p30 on the IFN innate response likely favors viral persistence in immune competent hosts.

#### 6.4.2. The p13 Protein

The viral protein p13 is produced from *orf-II* by a singly-spliced mRNA corresponding to the carboxy-terminal portion of p30 [71,73,75]. Using confocal microscopy and co-localization analyses with cellular compartment markers, electron microscopy, and biochemical fractionation, p13 was determined to localize predominantly to the inner mitochondrial membrane [202,203,204]. Several studies have shown that p13 alters mitochondrial function by increasing potassium influx, which in turn activates the electron transport chain favoring reactive oxygen species (ROS) production [203,205,206]. ROS are powerful second messengers that regulate multiple signal transduction pathways. Depending on their levels, ROS may favor cell proliferation, neoplastic transformation, or cell death. Observations made in isolated mitochondria found that p13 increased ROS production in several cell models, suggesting that p13 might contribute to an expansion of the pool of infected T-cells, but could possibly also trigger the apoptosis of transformed cells [207].

The effect of p13 on mitochondrial function could also affect the host immune response to the virus. Several studies have revealed important roles for mitochondria in immune responses [208]. By inducing cell death through mitochondrial pathways, p13 may trigger inflammatory responses in the host through the cyclic GMP-AMP synthase (cGAS)-stimulator of interferon genes (STING) signaling pathway [209]. Mitochondrial size and shape is controlled by the balance between mitochondrial fusion and fission [210]. This dynamic is connected to immune cell differentiation and activation. Naïve CD4^+^ T-cell activation induces a synchronized program of mitochondrial biogenesis and remodeling [211]. HTLV-1 infects myeloid cells altering the host innate immune responses [43,44,184,187,212,213]. It would be interesting to investigate the role p13 plays in affecting monocyte/macrophage and dendritic cell function. 

### 6.5. Viral and Host Factors That Regulate HTLV-1 Infectivity In Vivo

#### 6.5.1. Role of Viral Genes in HTLV-1 Infectivity

Unlike Tax, Rex and HBZ, the HTLV-1 regulatory genes p12, p8, p30, and p13 are not absolutely required for virus replication or immortalization of human primary T-cells in vitro [76,77,78]. The viral regulatory proteins are known to be expressed in infected individuals as antibodies, and cytotoxic T-lymphocytes to p12, p30, and p13 have been detected in patients [214,215,216]. The importance of the regulatory proteins to viral infection, dissemination, persistence, and clinical status has also been suggested in sequence analysis of the *orf-I* and *orf-II* regions in HTLV-1-infected individuals [74,139,206,217,218].

Several studies demonstrated that primary human T-cells immortalized with molecular clones lacking p12 or p30 grew less efficiently than the wild type molecular clone and are more dependent on IL-2 [78,79,80]. Early studies in the rabbit model suggested that p12, p13, and p30 might be important for viral infectivity [219,220,221]; however, it was recognized that these clones also have mutations in HBZ [222]. Subsequent studies re-investigating the role of p12 and p30 in molecular clones not affecting HBZ demonstrated that while HBZ, p12, and p30 were not essential for persistent infection in rabbits, these viral genes were critical for persistence in non-human primates [126,139]. The expression of *orf-I* is essential for infectivity in the macaque model and the requirement of *orf-I* for viral infectivity in macaques parallels HTLV-1 infectivity of dendritic cells in vitro [126]. No reversion of the single point mutation was observed in macaques, suggesting that virus-infected cells are eliminated very early following infection, precluding a sufficient round of viral replication to allow for the selection of virus revertant. Our further studies using HTLV-1 *orf-I* mutant viruses support the importance of p12/p8 expression and CD8^+^ cells in viral persistence [139]. In a humanized mouse model, we found that infection with wild type HTLV-1 virus resulted in polyclonal expansion of CD4^+^CD25^+^ T-cells. However, when mice were infected with virus ablated for orf-I expression, HTLV-1_p12KO_ infection only occurred after reversion of HTLV-1_p12KO_ back to wild type [127]. Similarly, using HTLV-2 in the rabbit model, the authors found that sequences in HTLV-2 corresponding to the p12 region in HTLV-1 are not necessary for infection, but confer increased replicative capacity in vivo [223]. In addition to *orf-I*, species specific requirements of *orf-II* and *hbz* for viral infectivity [126] suggest that non-human primates are the species of choice to test preventive vaccines for HTLV-1 that engage cellular immunity. 

#### 6.5.2. Role of NK, CD8, and Monocytes in HTLV-1 Infection

Increases in the HTLV-1 proviral load and persistent infection are likely linked with the virus’s ability to evade the host immune response. As stated above, p8 and p12 are dispensable for viral replication in vitro [76,77,126,224], but are essential for viral infectivity/persistence in vivo [126,139]. The p12 and p8 proteins counteract NK cells [143] and CD8^+^ cytotoxic T-cell (CTL) [139] responses in vitro and augment T-cell proliferation [79,225] and virus transmission [51,152,168]. The importance of *orf-I* expression for counteracting NK and CTL responses was validated in macaques by the depletion of either CD8 and NK cells (CD8/NK) or CD8 cells alone prior to virus exposure. HTLV-1 *orf-I* knockout virus is un-infectious in macaques, but following the depletion of CD8/NK, viral infectivity was restored and all animals were persistently infected with detectable mutated viral DNA in tissues [124]. Similarly, CD8/NK depletion accelerated virus infection after exposure to HTLV-1 wild type. While CD8 depletion alone accelerated the infectivity of HTLV-1 wild type, CD8 depletion, without the concomitant removal of NK cells, incompletely restored the infectivity of *orf-I* knockout HTLV-1 [124]. These data suggest that the innate function of NK cells is central for the immune control of HTLV-1 infectivity. Indeed, the frequency and function of NK cells is altered in HTLV-1 infection [226]. The frequency of spontaneous proliferation of NK cells correlates with proviral load in infected individuals [227]. Interestingly, NK cells may also play a role in chronic infection as passive transfer of amplified NK cells to a HTLV-1 patient with smoldering ATL resulted in complete remission [228]. 

Monocyte/macrophage depletion by clodronate prior to viral exposure to HTLV-1 wild type was associated with a faster seroconversion in all macaques, but antibody levels were not sustained, suggesting a possible role of monocytes in persistent infection [124]. The infectivity of *orf-I* knockout HTLV-1 was not restored by clodronate treatment prior to virus exposure. Interestingly, *orf-I* expression was associated with defective efferocytosis in part linked to its upregulation of CD47, the “don’t-eat-me” signal on infected cells [124]. These findings raise the possibility that *orf-I* expression by transiently protecting engulfed cells from degradation may facilitate the spread of virus by migratory efferocytosis to tissues. In addition, defective efferocytosis could create a durable and vicious inflammatory response that is unable to clear the virus by inducing further inflammation [229] and regulatory T-cell differentiation via the production of IL-10 and TGF-β [230]. Indeed, high levels of IL-10 and TGF-β and increased regulatory T-cell counts are hallmarks of HTLV-1 infection and may contribute to viral pathogenesis [46]. This study suggests that monocytes play a role early in infection by clearing infected cells. Alternatively, monocytes may provide an early viral reservoir important for maintaining viral persistence. Experiments which simultaneously deplete NK cells, CTLs, and monocytes in vivo are necessary to determine the role of monocytes in the early stages of infection.

## 7. Humoral Immunity

While the function of the viral regulatory proteins in modulating the T-cell response is actively being studied, little is known about the role these proteins play in modulating the HTLV-1 humoral response. In a study looking at a cohort of HTLV-1 exposed transfusion recipients, it was noted that antibodies to core, envelope and tax protein appeared within 30–60 days following primary HTLV-1 infection [231]. In most cases, the serum antibody titers correlate with the proviral load, but it is not known if high antibody titers contribute to protection or controlling the viral load [232,233]. 

Many viral vaccines are directed toward blocking virus entry into target cells. The HTLV-1 envelope (Env) protein is necessary for infection, highly immunogenic and the primary target of neutralizing antibodies [234]. Results from studies using passive immunization in animal models indicate that neutralizing antibodies could be protective. The administration of purified anti-HTLV-1 immunoglobulin from the plasma of seropositive individuals 24 h before HTLV-1 challenge protected cynomolgus monkeys from infection [235]. In addition, anti-HTLV-1 antibodies prevented viral transmission in NOD-SCID/γcnull mice [236] and rabbit models [237]. Furthermore, at birth, infants born to HTLV-1 positive mothers have detectable anti-HTLV-1 antibodies which decrease exponentially until most babies become seronegative by about nine months of age [238]. Interestingly, the duration of breastfeeding is an important risk factor associated with mother-to-child transmission, where longer duration of breastfeeding is associated with increased risk of viral transmission [238]. However, if this is due to neutralizing antibodies or increased repeated viral exposure remains unclear. In a study of 4 L from an HTLV-1 infected rabbit, neonates that were given anti-HTLV-1 hyperimmunoglobulin had a decreased risk of infection compared to untreated liters [239]. However, in rats, the infection of offspring by HTLV-1 positive mothers occurred at a higher rate in this model, which correlated with the proviral load. However, in this same model, passive administration of neutralizing antibodies did not prevent oral transmission [240]. Another complicating factor to consider is that although HTLV-1 Env is required for infection, viral cell-to-cell transmission through the VS, biofilms and cellular conduits is thought to shield the virus from antibodies [241]. 

As discussed above, NK cells play an important role in controlling viral persistence. Thus, eliciting anti-HTLV-1 antibodies may be important for clearance by antibody-dependent cellular cytotoxicity (ADCC). An early study examining ADCC and NK cell activity from newborns, infants and adults suggests that these activities can protect against the transmission of mother-to-child [242]. A more recent study found that a neutralizing anti-Env antibody, LAT-27, induced ADCC, eliminating Tax positive cells and can contribute to the control of infection [243]. A second study looking at NK cell activity in healthy carriers and HAM/TSP patients found that HAM/TSP patients had decreased frequencies of NK cells expressing CD16, the main receptor in the Fc-mediated antibody effector function inducing ADCC. This suggests that NK cells may prevent progression to HAM/TSP [226]. These results are consistent with the findings that ADCC activity was significantly reduced in HAM/TSP patients compared to asymptomatic carriers, due in part to a reduction in ADCC effector activity but not to a lack of anti-HTLV-1 ADCC antibodies [244].

## 8. Conclusions

**Figure 1:**
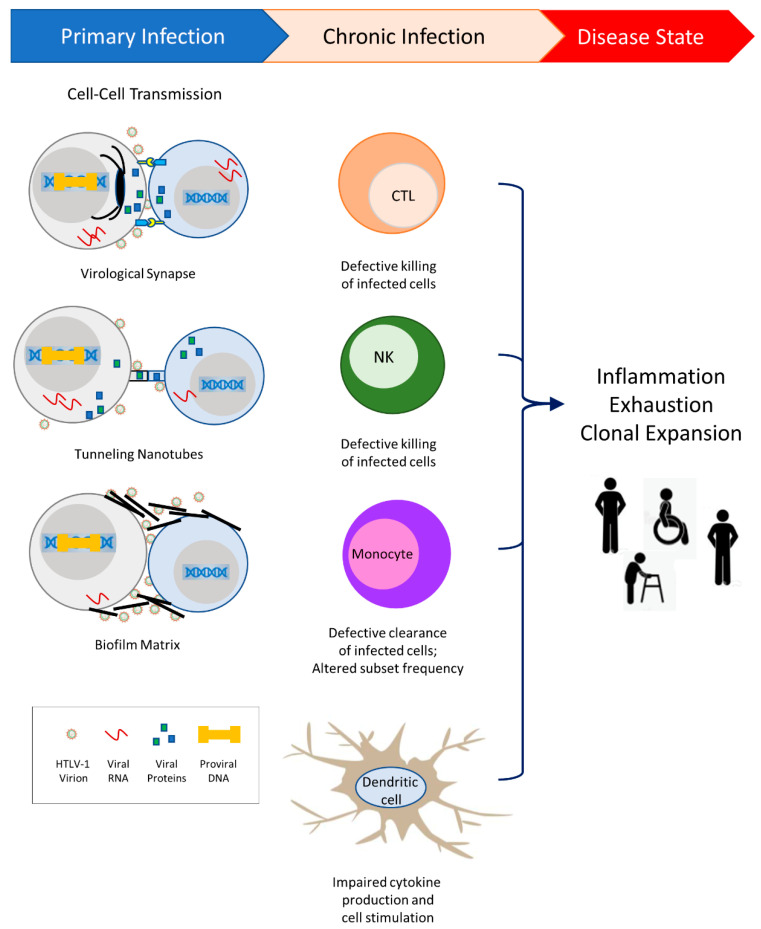
HTLV-1 transmission occurs primarily through cell-to-cell contact. Three modes of transmission have been demonstrated: virological synapse, cellular conduits called tunneling nanotubes, and biofilm matrices. HTLV-1 viral proteins enable the evasion of host immunity and contribute to alterations in the innate and adaptive immune responses. Altered responses to chronic HTLV-1 infection lead to inflammation and T-cell exhaustion, and allow clonal expansion of infected cells. While the majority of individuals remain asymptomatic, a subset of infected individuals will progress to diseases such as Adult T-cell Leukemia/Lymphoma, HTLV-1-associated myelopathy/tropical spastic paraparesis, HTLV-1-associated uveitis, bronchiectasis, rheumatoid arthritis, and infective dermatitis.

HTLV-1 counteracts host NK and CTL activity and usurps monocyte and dendritic cell immunity [43]. The continuous engagement of immune cells that fail to eradicate infection likely underlies the damaging chronic inflammation that ensues in a portion of HTLV-1-infected individuals (Figure 1). HTLV-1 infection has been reported to significantly alter dendritic cell function, increase the frequency of intermediate and non-classical (pro-inflammatory) monocytes, and decrease the frequency of classical monocytes that mediate the clearance of apoptotic cells and maintain tissue homeostasis [44]. The continuous but ineffective attempts of the immune system to clear the virus may result in exhaustion of both NK and CD8^+^ cells, as observed in infected individuals with high virus burdens [46,116,226,243,245,246,247,248].

Although an HTLV-1 preventative vaccine is feasible, no candidate vaccine has ever proceeded to clinical trial. Vaccine development efforts have used recombinant vaccinia virus vectors, protein immunization, DNA vaccine vectors, and peptide vaccines [249,250,251,252,253,254,255,256,257,258,259,260,261]. Collectively, these data suggest that an immune-based intervention based on vaccination alone is unlikely to be effective in the context of chronic HTLV-1 infection. With the current knowledge of HTLV-1 regulatory proteins, investigators should now consider targeting these pathways. For example, we recently showed in the rhesus macaque model that treatment of infected animals with the immunomodulator pomalidomide to target *orf-I*-mediated immune dysregulation caused reactivation of the virus, allowing its recognition by the host immune system [149]. Unfortunately, this response was short-lived, indicating that pomalidomide may not work as a single agent but could rather be used in combination therapy or in combination with vaccines. In addition, when HTLV-1-infected cells were treated in vitro with cytarabine, a therapeutic used in relapse/refractory AML [262], there was a reduction in tunneling-nanotubes induced by the viral p8 protein, reduced virus production, and reduced virus transmission [167]. Integrase inhibitors are another potential avenue to explore. Studies have shown that the integrase strand transfer inhibitors (INSTIs) raltegravir, bictegravir, and cabotegravir (FDA approved treatments for HIV-1) inhibited cell-free and cell-to-cell transmission of HTLV-1 in vitro [263,264,265,266]. Thus, INSTs should be considered in the treatment of HTLV-1, particularly for pre-exposure prophylaxis and in the prevention of mother to child transmission.

In addition, the data suggest that a preventive HTLV-1 vaccine should either prevent infection upfront or eliminate the virus very early on to avoid the establishment of a reservoir that host immunity is unable to clear. Given the HTLV-1 modes of transmission, virus vulnerability to neutralizing antibodies is uncertain. The engagement of less canonical host responses such as ADCC and efferocytosis, based on the ability of NK and monocytes to recognize and effectively dispose of infected cells, may be necessary for an HTLV-1 vaccine to prevent the establishment of infection.
